# Approaches to Assess Vitamin A Status in Settings of Inflammation: Biomarkers Reflecting Inflammation and Nutritional Determinants of Anemia (BRINDA) Project

**DOI:** 10.3390/nu10081100

**Published:** 2018-08-16

**Authors:** Leila M. Larson, Junjie Guo, Anne M. Williams, Melissa F. Young, Sanober Ismaily, O Yaw Addo, David Thurnham, Sherry A. Tanumihardjo, Parminder S. Suchdev, Christine A. Northrop-Clewes

**Affiliations:** 1 Department of Medicine, The University of Melbourne, Melbourne, VIC 3010, Australia; 2 Hubert Department of Global Health, Emory University, Atlanta, GA 30322, USA; junjie.guo@emory.edu (J.G.); melissa.young@emory.edu (M.F.Y.); sanober.rafiq.ismaily@emory.edu (S.I.); yaw.addo@emory.edu (O.Y.A.); 3 Nutrition Branch, CDC, Atlanta, GA 30329-4027, USA; nql3@cdc.gov; 4 Northern Ireland Centre for Food and Health, Ulster University, Coleraine, Co., BT52 1SA Londonderry, UK; di.thurnham@ulster.ac.uk; 5 Nutritional Sciences, University of Wisconsin-Madison, Madison, WI 53706, USA; sherry@nutrisci.wisc.edu; 6 Nutrition Branch, CDC, Atlanta, GA 30329-4027, USA; psuchde@emory.edu; 7 Department of Pediatrics, Emory University, Atlanta, GA 30322, USA; 8 Independent Public Health Nutrition Consultant, CB21 5JY Cambridge, UK; christinaclewes@btinternet.com

**Keywords:** Vitamin A, inflammation, retinol, retinol binding protein, infection

## Abstract

The accurate estimation of vitamin A deficiency (VAD) is critical to informing programmatic and policy decisions that could have important public health implications. However, serum retinol and retinol binding protein (RBP) concentrations, two biomarkers often used to estimate VAD, are temporarily altered during the acute phase response, potentially overestimating the prevalence of VAD in populations with high levels of inflammation. In 22 nationally-representative surveys, we examined (1) the association between C-reactive protein (CRP) or α1-acid glycoprotein (AGP) and retinol or RBP, and (2) how different adjustment approaches for correcting for inflammation compare with one another. In preschool age children (PSC) and school age children (SAC), the association between inflammation and retinol and RBP was largely statistically significant; using the regression approach, adjustments for inflammation decreased the estimated prevalence of VAD compared to unadjusted VAD (range: −22.1 to −6.0 percentage points). In non-pregnant women of reproductive age (WRA), the association between inflammation and vitamin A biomarkers was inconsistent, precluding adjustments for inflammation. The burden of VAD can be overestimated if inflammation is not accounted for, and the regression approach provides a method for adjusting retinol and RBP for inflammation across the full range of concentrations in PSC and SAC.

## 1. Introduction

Vitamin A deficiency (VAD) is an important public health problem worldwide, with implications for blindness and mortality, particularly in vulnerable populations of children and women [1,2]. Despite the important consequences of poor vitamin A status, its measurement in human populations is difficult. The gold standard assessment of an individual’s vitamin A status is the measurement of liver reserves, but this is not feasible in population surveys. Alternatively, serum retinol and retinol binding protein (RBP) concentrations are surrogate measures of vitamin A status commonly used in nutrition surveys [3,4]. However, both biomarkers are influenced by the presence of inflammation wherein concentrations transiently decrease during the acute phase response [5]. Therefore, the accurate estimation of VAD is difficult in contexts with high levels of inflammation.

During the inflammatory process there are rapid and large changes in the plasma concentrations of a number of nutrients used as nutritional biomarkers, including retinol and RBP [6]. In general, the metabolic changes that occur during inflammation are short-term, catabolic, and designed to inhibit and destroy the invading organisms. The production of acute phase proteins (APPs) is induced and regulated by cytokines. C-reactive protein (CRP) and α1-acid glycoprotein (AGP), two commonly measured APPs, are enhanced by the cytokines interleukin-6, interleukin-1, and tumor necrosis factor [7]. The time course of the APPs is related to their functions. There is an early rise in CRP (4–6 hours) concentrations, which respond acutely to the inflammatory stimulus with a much slower and longer response by AGP [8,9]. The acute phase response has a transient negative influence on retinol and RBP mainly because RBP is itself a negative APP [5], but also due to other factors [10]. A study by Louw et al. in healthy young adults who underwent uncomplicated orthopedic surgery found a significant transient decrease in blood concentrations of retinol and RBP, in addition to other nutritional biomarkers, corresponding with the acute phase response as monitored by CRP [11]. Specifically, plasma concentrations of RBP and retinol decreased significantly by 48 hours and corresponded with the changes in CRP. Concentrations returned to pre-operation levels after seven days. The transient decreases in vitamin A biomarkers can lead to overestimation of the prevalence of deficiency in populations with high levels of inflammation, resulting in estimates that are not reflective of the actual vitamin A status of the population. Yet to date, there are no universally accepted approaches to adjusting retinol and RBP for inflammation.

In response to this identified need, the Biomarkers Reflecting Inflammation and Nutritional Determinants of Anemia (BRINDA) project was formed in 2012 to analyze pooled data from population-based nutrition surveys and answer priority research questions around the assessment of nutritional biomarkers in settings of inflammation [12]. The current analysis builds on a previously published report from the BRINDA project examining effects of inflammation on RBP [13] and aims to summarize approaches to correct for inflammation when estimating VAD in children and women using both retinol and RBP. The focus of this paper is (1) to explore the associations between biomarkers of inflammation (CRP and AGP) and retinol and RBP in preschool age children (PSC), school age children (SAC) and non-pregnant women of reproductive age (WRA), and (2) to determine how different adjustment approaches for correcting for inflammation and for calculating the estimated prevalence of VAD compare with one another. This paper updates the previous BRINDA analyses by adding data from fourteen more surveys, by examining the associations between inflammation and retinol, and by examining the associations between inflammation, retinol, and RBP in a new population group, SAC.

## 2. Methods

The methods of the BRINDA project have previously been reported [14]. Briefly, surveys included in these analyses were nationally or regionally representative nutrition surveys that met the following inclusion criteria: (1) surveys were conducted after 2004, (2) target groups included PSC aged 6–59 months, SAC aged 5–15 years, or non-pregnant WRA aged 15–49 years, and (3) surveys measured retinol or retinol binding protein and one or more markers of inflammation (AGP and/or CRP). Data from 20 PSC, 6 SAC, and 14 WRA surveys were available for the present analysis.

Venous or capillary blood was collected from children and women, and plasma or serum was stored at −20 °C until analysis. Retinol was measured by high performance liquid chromatography and RBP by sandwich enzyme-linked immunosorbent assay (ELISA) [15]. CRP and AGP concentrations were assessed using sandwich ELISA in Bangladesh, Cambodia, Cameroon, Côte d’Ivoire, Kenya, Laos, Liberia, Papua New Guinea, and the Philippines, and by ELISA in Azerbaijan, Malawi, and Vietnam. CRP was measured using immunoassay in Afghanistan and Pakistan, using turbidimetry in Colombia and the United States, and using nephelometry in Ecuador and Mexico. In the United Kingdom, high sensitivity CRP was measured on a Siemens/DADE automated analyzer. AGP was measured using turbidimetry in Afghanistan, Mongolia, Nigeria, Nicaragua, and Pakistan.

Consistent with previously published BRINDA work on vitamin A [13] or WHO guidance [4], RBP or retinol concentrations <0.7 μmol/L were used to define VAD, respectively. The retinol:RBP relationship was assumed to be 1:1 [4,16], as only two surveys (Malawi and Cameroon) measured retinol and RBP in a subset of the same individuals. Based on the WHO classification, a VAD prevalence of ≥20% was defined as a severe public health problem, 10–19% as moderate, and 2–9% as mild [4]. Inflammation was defined as a CRP concentration >5 mg/L and/or AGP concentration >1 g/L [15,17].

All statistics were calculated with the use of SAS 9.4 software (SAS Institute, Cary, NC, USA). Correlations between RBP, retinol, CRP, and AGP concentrations were calculated using Spearman’s coefficient. The Taylor linearization method was used to obtain unbiased estimates that incorporated the sampling weight, strata, and cluster (as applicable) when analyzing individual surveys. The prevalence of VAD was estimated without any adjustments to retinol or RBP and is referred to as an unadjusted estimate. Adjustments for inflammation were made in PSC and SAC only because associations between retinol or RBP and CRP or AGP in WRA were inconsistent and often not significant. The following three approaches, explained in detail elsewhere [14], were used to adjust retinol and RBP for inflammation: exclusion, Correction Factor (CF), and regression correction (RC). The exclusion approach excluded observations that had elevated CRP or AGP concentrations, and the prevalence of estimated VAD was calculated with the remaining individuals. CFs were calculated based on internal survey-specific data [termed the Internal CF (ICF)] using a 4-group inflammation adjustment model of a reference (both CRP ≤ 5 mg/L and AGP ≤ 1 g/L), incubation (CRP > 5 mg/L and AGP ≤ 1 g/L), early convalescence (both CRP > 5 mg/L and AGP > 1 g/L), and late convalescence (CRP ≤ 5 mg/L and AGP > 1 g/L) similar to Thurnham et al.’s method [17]. ICFs were applied to individuals’ retinol and RBP concentrations based on their phase of inflammation. The RC approach uses linear regression to adjust retinol and RBP by the concentration of CRP and/or AGP on a continuous scale using internal survey-specific data [termed the Internal RC (IRC)]. Briefly, the adjusted retinol (or RBP) equation was calculated by subtracting the influence of CRP and AGP as follows:

Retinol_adjusted_ = retinol_unadjusted_ − β_1_(CRP_obs_ − CRP_ref_) − β_2_(AGP_obs_ − AGP_ref_) (1)

Depending on available data, CRP and/or AGP can be included in the model. β1 is the CRP regression coefficient and β2 is the AGP regression coefficient, obs is the observed value, and ref is the reference value (maximum value of the lowest CRP or AGP decile using either (1) the combined BRINDA data [14] reference values of CRP = 0.10, AGP = 0.59 for PSC for surveys, or (2) internal survey data reference values for SAC because of the limited amount of surveys; internal reference values for Ecuador PSC were used because the CRP assay limit of detection was >0.1 mg/L. CRP, AGP, and retinol were all ln transformed. The correction was only applied to individuals with ln-CRP_obs_ > ln-CRP_ref_ or ln-AGP_obs_ > ln-AGP_ref_ to avoid over-adjustments [14]. Additional information on the inflammation adjustment approaches, including statistical code, can be found on the BRINDA website (www.brinda-nutrition.org).

## 3. Results

### 3.1. Participant Characteristics

In PSC, prevalence of elevated CRP ranged from 7.3% in Bangladesh to 40.4% in Côte d’Ivoire, with a median of 14.3%; elevated AGP ranged from 21.2% in the Philippines to 64.5% in Côte d’Ivoire, with a median of 35.8% (Table 1). In SAC, elevated CRP ranged from 4.3% in Bangladesh to 16.2% in Malawi, with a median of 7.2%; elevated AGP was 15.4% in Bangladesh and 32.9% in Malawi. In WRA, elevated CRP ranged from 5.7% in Bangladesh to 25.7% in the United States, with a median of 13.0%; elevated AGP was between 7.2% in Cameroon and 33.5% in Cambodia, with a median of 17.3%. (Table 1). 

### 3.2. Relation between RBP, Retinol, and Inflammation

**Figure 1:**
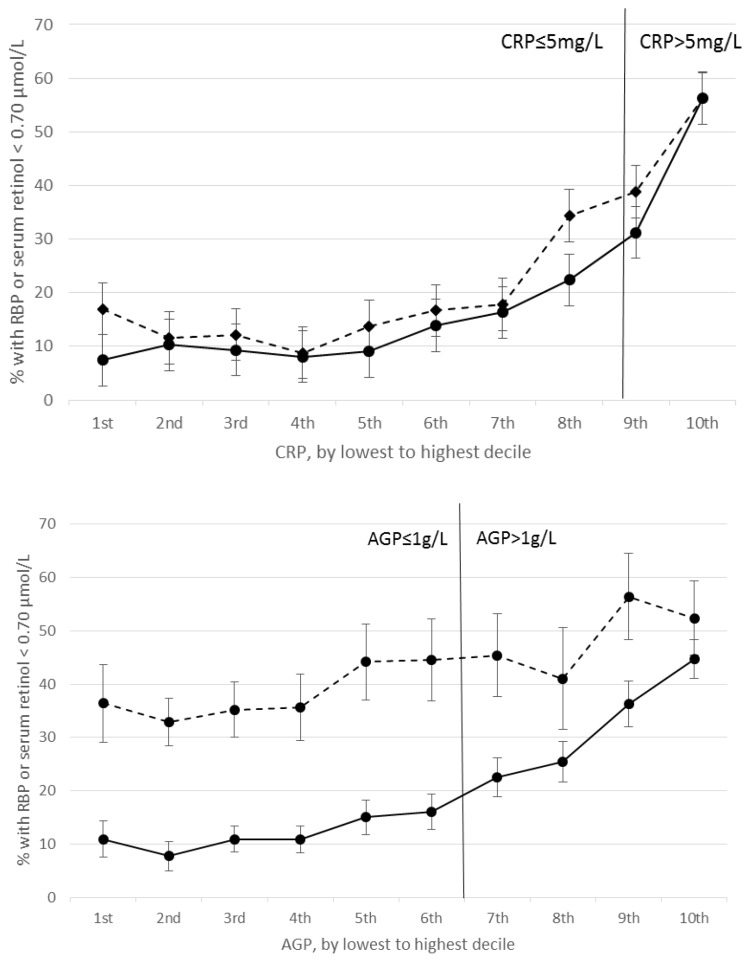
Estimated prevalence (95% CI) of vitamin A deficiency in preschool age children by C-reactive protein (CRP) (**top**) and α1-acid glycoprotein (AGP) (**bottom**) deciles. Top figure: solid line represents prevalence of retinol binding protein (RBP) < 0.70 μmol/L (*n* = 11,605), dotted line represents prevalence of retinol < 0.70 μmol/L (*n* = 9798). Bottom figure: Solid line represents prevalence of RBP < 0.70 μmol/L (*n* = 11,605), dotted line represents prevalence of retinol < 0.70 μmol/L (*n* = 10,055). AGP, α1-acid glycoprotein; CRP, C-reactive protein; RBP, retinol binding protein.

**Figure 2:**
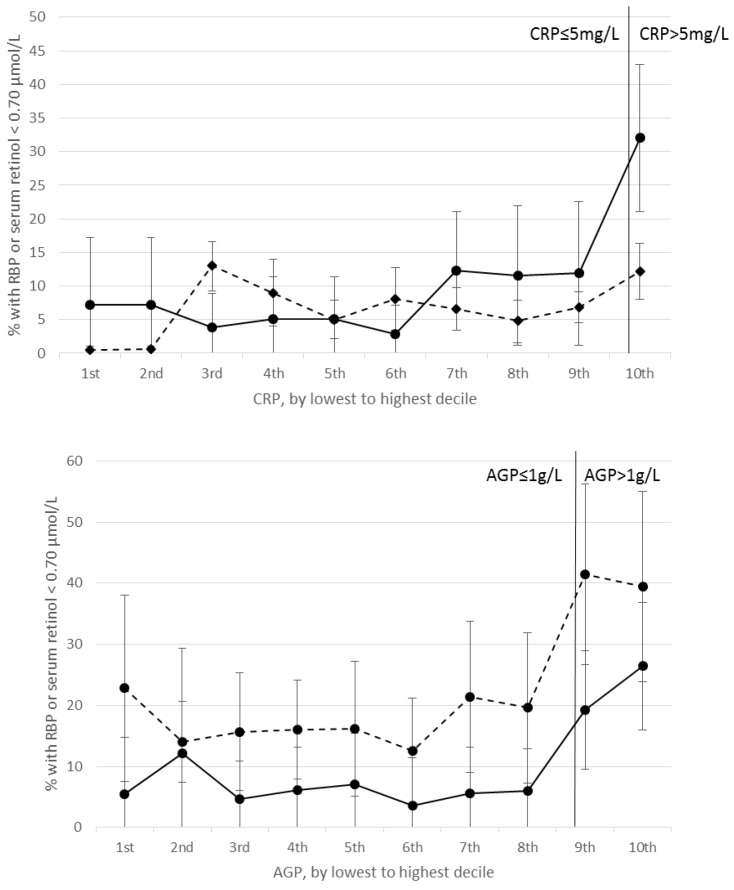
Estimated prevalence (95% CI) of vitamin A deficiency in school age children by CRP (**top**) and AGP (**bottom**) deciles. Top figure: solid line represents prevalence of RBP < 0.70 μmol/L (*n* = 750), dotted line represents prevalence of retinol < 0.70 μmol/L (*n* = 11,341). Bottom figure: Solid line represents prevalence of RBP < 0.70 μmol/L (*n* = 750), dotted line represents prevalence of retinol < 0.70 μmol/L (*n* = 1,271). AGP, α1-acid glycoprotein; CRP, C-reactive protein; RBP, retinol binding protein.

Correlations between RBP and CRP and between retinol and CRP were negative and significant in nearly all PSC and SAC surveys (Supplemental Table S1). RBP concentrations were positively correlated with CRP and AGP in Cambodian PSC. Excluding this survey, correlation coefficients between RBP or retinol and CRP in PSC and SAC ranged from −0.09 (United States) to −0.51 (Kenya 2010). Correlations between RBP and AGP and between retinol and AGP were negative and significant in PSC and SAC, except for in Pakistani PSC where the correlation was non-significant; significant negative correlations ranged from −0.14 (Bangladesh) to −0.45 (Kenya 2010) (Supplemental Table S1). Correlations below and above CRP = 5 mg/L and AGP = 1 g/L were generally consistent (data not shown). In both PSC and SAC, the estimated prevalence of VAD increased with increasing CRP and AGP deciles; larger increases in VAD are seen beyond the 4^th^ decile of CRP and AGP in PSC, and beyond the 9th decile in SAC (Figure 1 and Figure 2). Across the range of CRP and AGP concentrations, particularly in PSC, but also in SAC at higher CRP and AGP concentrations, estimated VAD increased with increasing inflammation above and below the cutoffs used to define inflammation (CRP = 5 mg/L and AGP = 1g/L).

In WRA, many surveys had mixed results and did not show a significant correlation between retinol or RBP and CRP, nor between retinol or RBP and AGP (Supplemental Table S1). For example, two of the seven surveys reported a positive significant correlation between RBP and CRP, and three of the seven surveys reported a positive significant correlation between RBP and AGP. Likewise, for correlations between serum retinol and CRP, three of the seven surveys indicated a significant positive correlation, one of the seven indicated a significant negative correlation and the remainder had non-significant correlations. Given the inconsistencies in the data, there was insufficient evidence to support the need for inflammation adjustment among WRA.

### 3.3. Unadjusted prevalence of VAD in PSC, SAC, and WRA

In PSC, unadjusted estimated prevalence of VAD (retinol or RBP < 0.7 μmol/L) ranged from 1.9% in Nigeria to 52.3% in Pakistan (Supplemental Table S2). In SAC, unadjusted estimated VAD ranged from 0.6% in the United States to 23.3% in Bangladesh. In WRA, unadjusted estimated prevalence of VAD ranged from 0.3% in the United States to 39.1% in Pakistan.

Adjustments for inflammation were not performed in WRA because associations between RBP, retinol, and CRP and AGP were inconsistent and of mixed statistical significance. Similar relationships between CRP, AGP, and RBP were described in previous BRINDA findings [13]. Further, unlike in children where the estimated prevalence of VAD decreased with increasing inflammation, in WRA, the prevalence of VAD varied across the range of CRP and AGP deciles (Supplemental Figure S1).

### 3.4. Estimated prevalence of VAD in PSC and SAC adjusting for inflammation

In PSC, the exclusion approach resulted in a sample size loss of 10–69%. Given Pakistani PSC had a poor correlation between retinol and AGP, and Cambodian PSC had positive correlations between RBP and CRP and AGP, no adjustments for inflammation were made in these two surveys. In all other surveys, excluding individuals with elevated CRP and AGP resulted in an estimated prevalence of VAD that was 12.0 absolute median percentage points (pps) lower than unadjusted VAD (range −18.0 to −5.5 pps) using RBP, and 6.6 pps lower (range −8.3 to −4.9 pps) using retinol (Table 2). The ICF approach resulted in a median decrease of 10.1 pps (range: −13.6 to −5.1 pps) using RBP, and 4.5 pps (−6.1 to −2.8 pps) using retinol. Lastly, using the IRC approach resulted in a prevalence of estimated VAD that was 16.4 median pps lower than unadjusted VAD (range: −22.1 to −6.0 pps) using RBP, and 13.1 median pps lower (range: −15.9 to −10.2 pps) using retinol. In the SAC surveys, as in PSC surveys, out of all the approaches, using the regression approach resulted in the largest median decrease in estimated VAD compared to unadjusted VAD (8.8 pps using RBP and 6.9 pps using retinol) (Table 2).

## 4. Discussion

In 20 surveys of PSC and six surveys of SAC from Asia, Sub-Saharan Africa, and North, Central, and South America, retinol and RBP concentrations were significantly negatively correlated with both CRP and AGP concentrations, except in two surveys. These associations were consistent among PSC and SAC with CRP and AGP concentrations above and below the standard cutoffs [15,17] used to define inflammation. On the other hand, in 14 surveys in non-pregnant WRA, retinol and RBP were not consistently negatively associated with biomarkers of inflammation. The inconsistent associations between biomarkers of vitamin A and inflammation in WRA could be a function of the women’s overall lower levels of inflammation, or other factors such as obesity and apo-RBP production [18]. This paper expands on previous BRINDA analyses [13] by showing that retinol behaves similarly to RBP in terms of its association with inflammation in PSC and SAC, but not in WRA. Associations between these biomarkers of vitamin A and inflammation were weaker in SAC compared to PSC, although the number of surveys in SAC is smaller. Adjustments for inflammation in PSC and SAC led to relatively lower decreases in estimated VAD when using retinol vs RBP, particularly in surveys with only one biomarker of inflammation.

Adjusting for inflammation decreased the estimated prevalence of VAD compared to unadjusted VAD. Our analyses indicated that adjusting retinol and RBP concentrations using the regression approach led to a substantive decrease in estimated VAD by a median of 13.1–16.4 pps in PSC, depending on the vitamin A biomarker, and 6.9–8.8 pps in SAC. Median decreases in surveys measuring only one inflammation biomarker were smaller than those calculated for surveys measuring both CRP and AGP. Correcting for both inflammatory biomarkers is important to account for the full timeframe of the inflammation, from acute to chronic, and adjusting for just one inflammatory biomarker may be insufficient. Further, median decreases in the estimated prevalence of VAD were larger in PSC than in SAC, likely due to the lower levels of inflammation in SAC (in PSC, median prevalence of elevated CRP was 14.3% and median elevated AGP was 35.8%; in SAC, median prevalence of elevated CRP was 7.2% and median elevated AGP was 24.2%).

In PSC and SAC, 14 of 26 surveys estimated an unadjusted VAD prevalence that indicated a severe public health problem. In seven of these surveys, the public health significance of VAD dropped from severe to mild after accounting for inflammation, and in four other surveys, the degree dropped from severe to moderate. It is important to note that these WHO categories were based on unadjusted VAD. However, the differences observed in estimated VAD prevalence before and after adjustment for inflammation have important consequences for vitamin A programs. In some settings, combinations of interventions exist: vitamin A fortification of centrally processed staple foods, point-of-use fortification including vitamin A, and vitamin A supplementation. Accurate estimation of the vitamin A status of a population is important when designing and implementing intervention programs to reduce VAD, particularly because of the potential negative consequences from vitamin A excess [19,20,21]. Further, in order to track progress of an intervention on the vitamin A status of a population, appropriate methodological comparisons over time are needed. Our results indicate the importance of accounting for inflammation in order to make more accurate conclusions on the VAD prevalence changes of a population over time.

The regression coefficients calculated in PSC from the 20 surveys included are heterogeneous. With more retinol and RBP data from children and a better understanding of the potential confounders of the association between retinol or RBP and inflammation, there may be an opportunity for universal regression coefficients to adjust for CRP and AGP. The need for additional data is particularly true for surveys of SAC with measures of both CRP and AGP, so as to examine the association between RBP and retinol with inflammation more fully. As suggested by Thurnham 2017 [22], until there is a unified formula for the regression approach, the CF approach is simpler to perform statistically. That said, the BRINDA project has macros available for use with several statistical software packages. However, both the CF and regression techniques for accounting for inflammation are limited when assessing survey data because the true vitamin A status, based on a liver biopsy, is unknown. One potential benefit of the regression approach, as demonstrated through the numerous surveys included here, is the ability to account for inflammation across the full range of CRP and AGP concentrations, including concentrations of CRP and AGP below the established cutoffs, an advantage the CF approach lacks. The regression approach results in a median decrease in estimated VAD from the unadjusted prevalence of 2–9 pps more than the CF approach.

A limitation of our analysis is the assumption of a 1:1 ratio for retinol:RBP; however, we did not have the data to confirm this ratio in the majority of surveys. In the two surveys that measured RBP in all individuals in addition to retinol in a subset, the retinol:RBP ratio was calculated and confirmed that the relationship was not 1:1 (Cameroon: 0.85 in PSC, 0.89 in WRA; Malawi: 1.19 in PSC, 1.17 in SAC, 1.16 in WRA). We explored whether adjusting for inflammation in PSC and SAC using the IRC approach changed this ratio, but found very little difference in the ratios before and after adjustment (ratios after adjustment in Cameroon: 0.85 in PSC; Malawi: 1.13 in PSC, 1.15 in SAC). These results and the range of ratios found in the literature [23] suggest a complicated relationship between retinol and RBP. More research on the factors associated with the retinol: RBP ratio is needed, particularly if RBP is to be used as a surrogate for retinol to define population level VAD. 

Using a meta-analysis of pooled data from existing surveys limits our ability to establish the validity of the regression approach. In future research, it would be important to compare inflammation-adjusted prevalence estimates to those derived using a measure of vitamin A status not influenced by inflammation. For example, a micronutrient survey in Malawi reported no VAD using the modified relative dose response (MRDR), which was consistent with the VAD prevalence using retinol and RBP [24]. Rapid, affordable, and simple assessment tools for the accurate estimation of vitamin A status are needed. The MRDR test has been presented as an appropriate alternative method to more accurately measure vitamin A status compared to retinol or RBP in field settings [2,23]. The MRDR test is reflective of liver vitamin A stores and is not as influenced by inflammation as compared to retinol or RBP [2,25]. Other research needs include investigating whether and how the cause of inflammation or type of infection may differentially influence the association between inflammation and retinol and RBP, and assessing the difference between acute and chronic inflammation on biomarkers of vitamin A status. 

## 5. Conclusions

In summary, associations between retinol or RBP and CRP and AGP are consistently negative in PSC and SAC, and these associations exist above and below the thresholds typically used to define inflammation. The regression approach provides a method for adjusting retinol and RBP for inflammation across the full range of concentrations, in order to estimate the prevalence of VAD in populations with varying levels of inflammation. The burden of VAD can be overestimated if inflammation is not accounted for and this finding requires additional research to validate in order to inform vitamin A programs. 

## Figures and Tables

**Table 1:** Age, inflammation, vitamin A status, and malaria among preschool age children, school age children, and women of reproductive age, Biomarkers Reflecting Inflammation and Nutritional Determinants of Anemia (BRINDA) project.

Country, year	*n*	Age (Months) Mean (Min, Max)	RBP (μmol/L) Median (95% CI)	Serum Retinol (μmol/L) Median (95% CI)	CRP > 5mg/L Percent (95% CI)	AGP > 1g/L Percent (95% CI)	CRP > 5mg/L or AGP > 1g/L Percent (95% CI)
**Preschool Age Children**							
Afghanistan, 2013	657	29.1 (6, 58)		0.70 (0.67, 0.73)	10.1 (6.9, 13.4)	23.6 (19.3, 27.9)	25.7 (20.8, 30.5)
Azerbaijan, 2013	1053	35.6 (6, 59)	1.01 (0.98, 1.03)		8.1 (6.0, 10.1)	29.9 (26.2, 33.5)	30.9 (27.1, 34.6)
Bangladesh, 2010	1493	8.3 (6, 11)	0.88 (0.87, 0.90)		14.3 (11.8, 16.7)	33.4 (29.9, 36.9)	35.8 (32.2, 39.5)
Bangladesh, 2012	458	36.5 (6, 59)		0.85 (0.81, 0.89)	7.3 (3.2, 11.5)	28.5 (22.6, 34.4)	29.0 (23.1, 34.9)
Cambodia, 2014	665	35.9 (6, 60)	1.32 (1.23, 1.40)		10.0 (7.3, 12.8)	36.2 (29.5, 42.9)	38.3 (30.6, 46.0)
Cameroon, 2009^1^	774	31 (12, 60)	0.84 (0.82, 0.87)	0.70 (0.62, 0.77)	37.5 (32.7, 42.3)	39.3 (33.7, 45.0)	48.3 (43.1, 53.5)
Colombia, 2010	3794	37.6 (12, 59)		0.85 (0.83, 0.87)	18.8 (17.1, 20.6)		
Côte d’Ivoire, 2007	733	31.7 (6, 59)	0.89 (0.86, 0.92)		40.4 (36.5, 44.3)	64.5 (60.3, 68.6)	67.5 (63.8, 71.3)
Ecuador, 2012	2017	30.8 (6, 59)		0.88 (0.86, 0.90)	12.5 (10.1, 14.9)		
Kenya, 2007	888	19.9 (6, 36)	0.87 (0.85, 0.90)		27.8 (23.9, 31.7)	64.2 (60.2, 68.2)	66.0 (61.9, 70.1)
Kenya, 2010	843	21.4 (6, 35)	0.84 (0.81, 0.87)		34.2 (29.6, 38.7)	60.7 (56.0, 65.4)	61.9 (57.2, 66.6)
Liberia, 2011	1434	19.9 (6, 36)	0.85 (0.82, 0.88)		29.5 (26.5, 32.5)	56.2 (52.5, 60.0)	59.1 (55.6, 62.7)
Mongolia, 2006 ^2^	202	20 (7, 36)		0.79 (0.74, 0.83)		26.2 (20.2, 32.3)	
Malawi, 2016 ^1^	1084	32.5 (6, 59)	0.86 (0.82, 0.90)	1.00 (0.82, 1.19)	23.7 (18.6, 28.7)	55.9 (50.3, 61.5)	57.0 (51.2, 62.7)
Mexico, 2012	2512	39.1 (12, 60)		0.93 (0.91, 0.96)	11.6 (9.3, 14.0)		
Nigeria, 2005	1420	33.4 (6, 60)		1.22 (1.17, 1.26)		24.0 (20.5, 27.5)	
Pakistan, 2011	7318	27.3 (6, 59)		0.67 (0.65, 0.69)		35.3 (33.8, 36.8)	
Papua New Guinea, 2005	871	31.4 (6, 60)	0.87 (0.84, 0.90)		31.6 (27.2, 36.0)	54.1 (49.4, 58.9)	57.0 (52.6, 61.5)
Philippines, 2011	1767	15 (6, 24)	1.03 (1.01, 1.05)		13.9 (11.6, 16.2)	21.2 (17.7, 24.6)	26.0 (22.4, 29.5)
Vietnam, 2010	360	37.3 (10, 60)		1.16 (1.11, 1.21)	12.8 (9.7, 15.8)		
**School Age Children**							
Bangladesh, 2010	1271	9.4 (6, 14)		0.86 (0.83, 0.88)	4.3 (2.0, 6.6)	15.4 (12.1, 18.7)	16.0 (12.6, 19.4)
Ecuador, 2012	3281	7.6 (5, 15)		0.92 (0.90, 0.94)	7.7 (5.7, 9.7)		
Malawi, 2016 ^1^	750	9.5 (5, 15)	0.98 (0.94, 1.01)	1.11 (1.02, 1.19)	16.2 (12.3, 20.2)	32.9 (28.6, 37.3)	35.0 (30.2, 39.8)
Mexico, 2012	3144	8.6 (5, 12)		1.17 (1.15, 1.18)	7.7 (6.4, 9.0)		
United Kingdom, 2014	556	9.9 (5, 14)		1.16 (1.12, 1.19)	4.6 (2.2, 6.9)		
United States, 2006	3089	10.8 (6, 15)		1.33 (1.32, 1.35)	6.6 (5.2, 8.1)		
**Women of Reproductive Age**							
Afghanistan, 2013	1046	30.9 (15, 49)		1.13 (1.07, 1.19)	12.8 (10.4, 15.2)	11.6 (8.8, 14.4)	19.3 (15.8, 22.8)
Azerbaijan, 2013	2656	32.1 (15, 50)	1.46 (1.44, 1.49)		13.2 (11.3, 15.1)	31.3 (29, 33.6)	34.5 (32.0, 36.9)
Bangladesh, 2012	897	29.7 (15, 49)		1.12 (1.07, 1.16)	5.7 (3.2, 8.2)	12.8 (8.9, 16.6)	16.7 (12.4, 20.9)
Cambodia, 2014	705	30.2 (16, 49)	1.96 (1.74, 2.18)		9.5 (7.1, 11.8)	33.5 (24.9, 42.2)	36.7 (27.3, 46.0)
Cameroon, 2009 ^1^	751	27.2 (15, 48)	1.44 (1.40, 1.48)	1.24 (1.16, 1.32)	17.8 (14.8, 20.7)	7.2 (5.1, 9.3)	19.7 (16.6, 22.9)
Côte d’Ivoire, 2007	816	27.6 (15, 48)	1.49 (1.44, 1.54)		19.7 (16.5, 22.8)	26.9 (23.5, 30.4)	33.7 (29.6, 37.9)
Ecuador, 2012	5979	33.2 (19, 49)		1.27 (1.25, 1.28)	19.0 (17.1, 21.0)		
Liberia, 2011	1875	28.6 (15, 50)	1.33 (1.30, 1.36)		14.3 (12.1, 16.4)	10.4 (8.7, 12.2)	18.5 (16.2, 20.8)
Malawi, 2016 ^1^	753	28.1 (15, 49)	1.39 (1.34, 1.44)	1.39 (1.26, 1.53)	7.5 (5.1, 9.9)	10.7 (7.5, 13.8)	13.0 (9.6, 16.4)
Pakistan, 2011	5929	30.8 (16, 49)		0.84 (0.81, 0.88)	12.0 (11.0, 13.0)	24.1 (22.7, 25.6)	31.6 (30.0, 33.1)
Papua New Guinea, 2005	749	29.1 (15, 49)	1.61 (1.57, 1.66)		10.0 (7.5, 12.5)	21.8 (18.1, 25.6)	24.8 (21.0, 28.7)
United Kingdom, 2014	875	34.6 (15, 49)		1.61 (1.55, 1.68)	15.8 (12.5, 19.1)		
United States, 2006	3145	33.5 (15, 50)		1.74 (1.72, 1.77)	25.7 (23.5, 27.8)		
Vietnam, 2010	1434	32.3 (15, 49)		1.62 (1.59, 1.65)	6.7 (5.5, 7.9)		

1 Cameroon and Malawi’s serum retinol was measured in a subgroup (Cameroon *N* = 115 for preschool children and *N* = 104 for women; Malawi *N* = 73 for preschool children, *N* = 84 for school children, and *N* = 89 for women.); 2 Mongolia did not apply complex survey design.

**Table 2:** Summary of change in estimated vitamin A deficiency (VAD) by adjustment method across surveys, preschool age children, and school age children, BRINDA project.

Approach	Absolute Median (Range) Percentage Point (pp) Difference for Surveys That Measured both CRP and AGP		Absolute Median (Range) Percentage Point (pp) Difference for Surveys That Measured either CRP or AGP	
	RBP ^1^	Retinol	RBP ^1^	Retinol
**Preschool Children**				
Sample size (No. of surveys)	10	2	-	6
Exclusion	−12.0 pp (−18.0 pp, −5.5 pp)	−6.6 pp (−8.3 pp, −4.9 pp)	-	−3.4 pp (−7.2 pp, −0.2 pp)
ICF	−10.1 pp (−13.6 pp, −5.1 pp)	−4.5 pp (−6.1 pp, −2.8 pp)	-	−3.6 pp (−4.9 pp, −0.5 pp)
IRC	−16.4 pp (−22.1 pp, −6.0 pp)	−13.1 pp (−15.9 pp, −10.2 pp)	-	−6.4 pp (−9.9 pp, −1.1 pp)
**School-Aged children**				
Sample size (No. of surveys)	1	1	-	4
Exclusion	−6.6 pp	−4.0 pp	-	−0.5 pp (−2.0 pp, −0.3 pp)
ICF	−7.1 pp	−3.0 pp	-	−0.5 pp (−1.6 pp, −0.1 pp)
IRC	−8.8 pp	−6.9 pp	-	−0.8 pp (−3.9 pp, −0.2 pp)

1. All countries with RBP data measured both CRP and AGP. ICF, Internal Correction; IRC, Internal Regression Correction; CRP, C-reactive protein; AGP, α1-acid glycoprotein; RBP, retinol binding protein; VAD, vitamin A deficiency; BRINDA, Biomarkers Reflecting Inflammation and Nutritional Determinants of Anemia.

## Supplementary Materials

The following are available online at http://www.mdpi.com/2072-6643/10/8/1100/s1, Figure S1: Estimated prevalence (95% CI) of vitamin A deficiency in women of reproductive age by CRP (top) and AGP (bottom) deciles, Table S1: Correlations between serum retinol or RBP, and inflammation among preschool children, school age children, and women of reproductive age, BRINDA project, Table S2: Estimated prevalence of vitamin A deficiency (serum retinol or RBP < 0.7µmol/L) unadjusted and after adjustment in preschool age children, school age children, and women of reproductive age BRINDA project.
