# Supplementary material for: Approaches to Assess Vitamin A Status in Settings of Inflammation: Biomarkers Reflecting Inflammation and Nutritional Determinants of Anemia (BRINDA) Project
Source: Nutrients. 2018 Aug 16;10(8):1100. doi: 10.3390/nu10081100 (PMC6115742; doi:10.3390/nu10081100)
Supplement: Supplementary file 1 [file nutrients-10-01100-s001.pdf]

Supplemental Table S1: Correlations between serum retinol or RBP, and inflammation among preschool children, school age children, and women of reproductive age, BRINDA project[1]

	RBP		Serum retinol		CRP
Country	CRP	AGP	CRP	AGP	AGP
PSC					
Afghanistan(2013)			-0.26**	-0.28**	0.59**
Azerbaijan(2013)	-0.37**	-0.22**			0.66**
Bangladesh(2010)	-0.37**	-0.27**			0.64**
Bangladesh(2012)			-0.32**	-0.31**	0.69**
Cambodia(2014)	0.09*	0.64**			0.59**
Cameroon(2009)	-0.41**	-0.32**			0.71**
Colombia(2010)			-0.17**		
Côte d'Ivoire(2007)	-0.47**	-0.39**			0.65**
Ecuador(2012)			-0.37**		
Kenya(2007)	-0.45**	-0.38**			0.66**
Kenya(2010)	-0.51**	-0.45**			0.76**
Liberia(2011)	-0.37**	-0.34**			0.65**
Malawi(2016)	-0.42**	-0.33**			0.64**
Mexico(2012)			-0.37**		
Mongolia(2006)				-0.24**	
Nigeria(2005)				-0.25**	
Pakistan (2011)				-0.01	
Papua New Guinea(2005)	-0.44**	-0.26**			0.69**
Philippines(2011)	-0.48**	-0.38**			0.75**
Vietnam(2010)			-0.26**		
SAC					
Bangladesh(2010)			-0.18**	-0.14**	0.59**
Ecuador(2012)			-0.23**		
Malawi(2016)	-0.39**	-0.37**			0.68**
Mexico(2012)			-0.13**		
United Kingdom (2014)			-0.11*		
United States (2006)			-0.09**		
WRA					
Afghanistan(2013)			-0.01	0.02	0.38**
Azerbaijan(2013)	0.15**	0.20**			0.63**
Bangladesh(2012)			0.11*	-0.16**	0.53**
Cambodia(2014)	0.22**	0.69**			0.40**
Cameroon(2009)	-0.15**	-0.05			0.57**
Côte d'Ivoire(2007)	-0.03	0.01			0.5**
Ecuador(2012)			-0.08**		
Liberia(2011)	-0.11**	0.06*			0.52**
Malawi(2016)	-0.10*	0			0.48**
Pakistan (2011)			0	0.05**	0.19**
Papua New Guinea(2005)	-0.17**	-0.02			0.53**
United Kingdom (2014)			0.02		
United States (2006)			0.09**		
Vietnam(2010)			0.07*		

1. **, p-value<0.001; *, p-value<0.05

Supplemental Table S2: Estimated prevalence of vitamin A deficiency (serum retinol or RBP < 0.7µmol/L) unadjusted and after adjustment in preschool age children, school age children, and women of reproductive age BRINDA project[1]

	RBP						Retinol					
Country	n	Unadjusted	ICF[2]	IRC[3]	n	Exclusion[4]	n	Unadjusted	ICF[2]	IRC[3]	n	Exclusion[4]
						Preschool age children						
Afghanistan(2013)							657	47.6 (42.1,53.1)	41.5 (36.8,46.2)	37.4 (32.2,42.7)	478	39.3 (33.1,45.5)
Azerbaijan(2013)	1053	12.2 (8.9,15.5)	7.1 (4.6,9.7)	6.1 (3.6,8.6)	756	5.8 (2.9,8.8)						
Bangladesh(2010)	1493	17.0 (14.4,19.7)	10.1 (7.6,12.6)	5.6 (3.6,7.5)	958	9.6 (6.8,12.4)						
Bangladesh(2012)							458	20.6 (14.1,27.1)	17.8 (11.7,24)	4.7 (2,7.3)	320	15.7 (7.6,23.8)
Cambodia(2014)[5]	665	9.9 (6.5,13.3)										
Cameroon(2009)	774	28.5 (24.3,32.7)	17.3 (13.9,20.6)	9.3 (7,11.7)	411	16.6 (12,21.2)						
Colombia(2010)							3794	23.9 (21.9,25.8)	20.5 (18.6,22.4)	17.6 (15.9,19.3)	3069	21.1 (19,23.2)
Côte d'Ivoire(2007)	733	24.0 (20.3,27.8)	12.1 (9.8,14.4)	2.7 (1.4,4)	227	8.7 (5.3,12)						
Ecuador(2012)							2017	22.4 (19.4,25.4)	18.4 (15.5,21.2)	15.9 (12.8,19)	1772	18.5 (15.6,21.5)
Kenya(2007)	888	23.0 (19.1,26.8)	11.1 (8.5,13.8)	5.5 (3.7,7.4)	302	6.3 (3.3,9.3)						
Kenya(2010)	843	29.5 (25.7,33.4)	15.9 (13.3,18.5)	7.4 (5.9,8.8)	321	11.5 (7.9,15.1)						
Liberia(2011)	1434	24.7 (21.2,28.1)	14.3 (11.9,16.8)	5.3 (3.8,6.8)	633	11.6 (8.4,14.8)						
Mongolia(2006)[6]							202	36.1 (29.5,42.8)	31.2 (24.8,37.6)	26.2 (20.3,32.3)	149	28.9 (21.6,36.1)
Malawi(2016)[4]	1084	23.0 (19.1,26.8)	16 (12.4,19.6)	7.7 (5.1,10.2)	444	13 (5.8,20.1)						
Mexico(2012)							2512	15.9 (13.4,18.4)	12.2 (10,14.4)	7.3 (5.7,8.9)	2253	11.3 (9.1,13.6)
Nigeria(2005)							1420	1.9 (1,2.7)	1.4 (0.7,2.1)	0.8 (0.3,1.3)	1083	0.7 (0.2,1.2)
Papua New Guinea(2005)	871	25.0 (20.8,29.3)	15.2 (12.2,18.1)	9.9 (7.7,12)	369	12.9 (8.9,16.9)						
Philippines(2011)	1767	6.9 (5.1,8.7)	1.8 (1.1,2.5)	0.9 (0.4,1.4)	1274	1.4 (0.8,1.9)						
Pakistan (2011)[5]							7318	52.3 (50.1,54.6)				
Vietnam(2010)							360	6.9 (4.3,9.6)	6.1 (3.7,8.6)	5.6 (3.1,8)	314	6.7 (3.8,9.5)
						School age children						

Bangladesh(2010)							1271	23.3 (18.9,27.7)	20.3 (16.6,24)	16.4 (12.7,20)	1089	19.3 (15.4,23.2)
Ecuador(2012)							3281	15.1 (12.5,17.6)	13.5 (11,16)	11.2 (9.2,13.1)	3060	13.1 (10.4,15.8)
Malawi(2016)	758	12.6 (9.1,16.2)	5.5 (2.6,8.3)	3.8 (1.6,6)	483	6.0 (3.5,8.5)						
Mexico(2012)							3144	2.3 (1.7,3)	1.7 (1.1,2.3)	1.4 (0.9,2)	2874	1.7 (1,2.3)
United Kingdom (2014)							556	1.9 (0.7,3.1)	1.6 (0.5,2.8)	1.2 (0.2,2.3)	536	1.6 (0.5,2.8)
United States (2006)							3089	0.6 (0.4,0.8)	0.5 (0.3,0.7)	0.4 (0.2,0.6)	2857	0.3 (0.1,0.5)
Women of reproductive age												
Afghanistan(2013)							1046	10.1 (7.5,12.7)				
Azerbaijan(2013)	2656	0.4 (0.1,0.7)										
Bangladesh(2012)							897	7.2 (3.7,10.7)				
Cambodia(2014)	705	2.9 (1.6,4.1)										
Cameroon(2009)	751	1.5 (0.6,2.4)					104	6.8 (1.6,12)				
Côte d'Ivoire(2007)	816	0.7 (0.2,1.3)										
Ecuador(2012)							5979	2.6 (1.5,3.7)				
Liberia(2011)	1875	2.3 (1.4,3.1)										
Malawi(2016)	753	3 (1.3,4.7)										
Papua New Guinea(2005)	749	0.6 (0,1.1)										
Pakistan (2011)							5929	39.1 (36.8,41.4)				
United Kingdom (2014)							875	1 (0,2.1)				
United States (2006)							3145	0.3 (0.1,0.6)				
Vietnam(2010)							1434	1.3 (0.7,1.9)				

1. Values are percent (95% CI); AGP, α1-acid glycoprotein; BCF, BRINDA Correction Factor; CRP, C-Reactive Protein; ICF, Internal Correction; IRC, Internal Regression Correction; Vitamin A deficiency defined as retinol binding protein or serum retinol < 0.70 µmol/L.
2. Country-specific internal correction factors - four stages of inflammation for RBP analyses:
   PSC: Azerbaijan: CRP=1.21 CRP+AGP=1.43 AGP=1.12; Bangladesh: CRP=1.31 CRP+AGP=1.27 AGP=1.11; Côte d'Ivoire: CRP=1.18 CRP+AGP=1.38 AGP=1.08; Cambodia: CRP=1.25 CRP+AGP=0.70 AGP=0.49; Cameroon: CRP=1.21 CRP+AGP=1.30 AGP=1.04; Kenya2007: CRP=1.28 CRP+AGP=1.42 AGP=1.10; Kenya2010: CRP=1.13 CRP+AGP=1.48 AGP=1.08; Liberia: CRP=1.10 CRP+AGP=1.34 AGP=1.09; Malawi: CRP=1.42 CRP+AGP=1.32 AGP=1.07; Papua New Guinea: CRP=1.25 CRP+AGP=1.30 AGP=1.03; Philippines: CRP=1.27 CRP+AGP=1.50 AGP=1.11
   SAC: Malawi: CRP=1.21 CRP+AGP=1.38 AGP=1.12
   Country-specific internal correction factors - four stages of inflammation for serum retinol analyses:

PSC: Afghanistan CRP=1.02 CRP+AGP=1.57 AGP=1.27; Bangladesh2012: CRP=1.63 CRP+AGP=1.24 AGP=1.03; Colombia: CRP=1.13; Ecuador CRP=1.26; Mexico2010 CRP=1.36; Vietnam CRP=1.12; Mongolia AGP=1.20; Nigeria AGP=1.12; Pakistan AGP=0.96
SAC: Bangladesh2012: CRP=1.11 CRP+AGP=1.47 AGP=1.09; Ecuador: CRP=1.21; Mexico2013: CRP=1.17; UK CRP=1.15; US: CRP=1.17

3. Survey-specific internal BRINDA regression correction (p-value) for RBP analyses:
PSC: Bangladesh: lnCRP=-0.051(<0.001) lnAGP=-0.041(ns); Cameroon: lnCRP=-0.054(<0.001) lnAGP=-0.115(<0.05); Côte d'Ivoire: lnCRP=-0.074(<0.001) lnAGP=-0.171(<0.001); Kenya 2007: lnCRP=-0.049(<0.001) lnAGP=-0.158(<0.001); Kenya 2010: lnCRP=-0.063(<0.001) lnAGP=-0.174(<0.001); Liberia: lnCRP=-0.051(<0.001) lnAGP=-0.199(<0.001); Papua New Guinea: lnCRP=-0.067(<0.001) lnAGP=0.038(ns); Philipines: lnCRP=-0.064(<0.001) lnAGP=-0.052(<0.05); Cambodia: lnCRP=-0.174(<0.001) lnAGP=0.659(<0.001); Malawi: lnCRP=-0.063(<0.001) lnAGP=-0.058(<0.05); Azerbaijan: lnCRP=-0.056(<0.001) lnAGP=-0.007(ns)
SAC: Malawi: lnCRP=-0.043(<0.001) lnAGP=-0.123 (<0.001);
Survey-specific internal BRINDA regression correction (p-value) for serum retinol analyses:
PSC: Afghanistan lnCRP=-0.048 (<0.001), lnAGP=-0.160 (<0.05); Bangladesh lnCRP=-0.062(<0.001), lnAGP=-0.147 (<0.05); Colombia lnCRP=-0.032(<0.001); Mexico lnCRP=-0.069 (<0.001); Mongolia lnAGP=-0.287 (<0.05); Nicaragua lnAGP=-0.220 (<0.001); Pakistan lnAGP=-0.023 (ns); Vietnam lnCRP=-0.043 (p<0.05)
SAC: Bangladesh2012: lnCRP=-0.073(<0.001) lnAGP=-0.036(ns); Ecuador: lnCRP= -0.147(<0.001); Mexico2012: lnCRP=-0.027(<0.001); UK: lnCRP= -0.069(<0.001); US: lnCRP= -0.023 (<0.001)
4. Vitamin A deficiency among the subset of samples with non-elevated inflammatory biomarkers.
5. No adjustments were made for Pakistan and Cambodia surveys, because of poor correlation between vitamin A measures and inflammation,
6. Mongolia did not apply complex survey design, so binomial proportion test was used to calculate 95% CI.

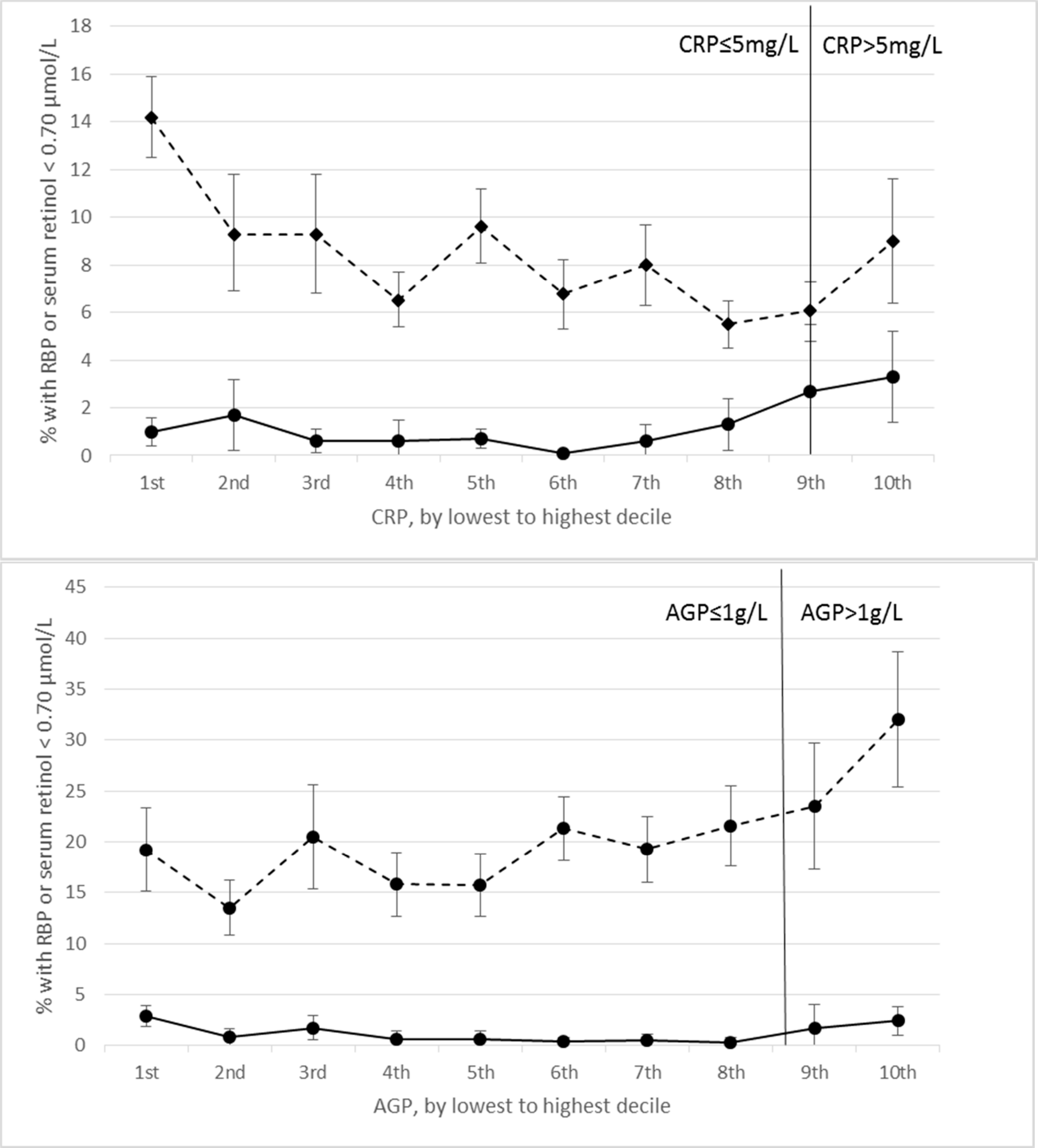

Supplemental Figure S1: Estimated prevalence (95% CI) of vitamin A deficiency in women of reproductive age by CRP (top) and AGP (bottom) deciles. Top figure: solid line represents prevalence of RBP<0.70 µmol/L (n= 8,305), dotted line represents prevalence of retinol<0.70 µmol/L (n= 19,305). Bottom figure: solid line represents prevalence of RBP<0.70 µmol/L (n= 8,305), dotted line represents prevalence of retinol<0.70 µmol/L (n= 7,872). AGP, α1-acid glycoprotein; CRP, C-reactive protein; RBP, retinol binding protein.
